# Campus-Wide Buy-In for an Age-Friendly University: One Goal and Many Paths

**DOI:** 10.1093/geroni/igaa057.1748

**Published:** 2020-12-16

**Authors:** Andrea Zakrajsek, Carrie Andreoletti

## Abstract

Recognizing a growing aging population around the world as well as the many benefits of engaging learners at any age in higher education institutions (Kressley & Huebschmann, 2002; Morrow-Howell, et al., 2019; Silverstein, Choi, & Bulot, 2001), the Age-Friendly University (AFU) international initiative offers a medium to support diversity and inclusion efforts based upon age. Dublin City University (DCU), along with Arizona State University (ASU) and Strathclyde University, developed 10 Age-Friendly University (AFU) principles which offer a guide for institutional commitment to age-diversity that can be realized through institutional goals, aims, and initiatives (DCU, n.d., Talmage, Mark, Slowely, & Knopf, 2016). Because of the non-prescriptive nature of these principles, universities endorsing them have opportunities to forge varied paths in the unified goal of age-friendliness. Presenters will share lessons learned from development of the AgeAlive collaborative hub to advance age-friendly research and community-based projects at Michigan State University, the value of cross-campus partnerships at the University of Hartford, the critical support provided by the Adult Learner Programs and Services office at Northern Kentucky University, and the intentional alignment of AFU efforts with administrative priorities at Eastern Michigan University, and journey from focusing on programs to embedding age-friendly practices throughout the institution at Arizona’s State University. Through the diverse paths these presenters used to obtain support for the AFU principles at their respective universities, participants who are just beginning their AFU journeys will learn actionable strategies for increasing age-friendliness at their own institutions.

